# Signet-ring cell carcinoma of the appendix with ganglioneuromatosis: a case report

**DOI:** 10.1186/s40792-022-01509-3

**Published:** 2022-08-05

**Authors:** Ayami Sato, Yu Sato, Nobuyuki Hiruta, Takashi Oshiro, Yutaka Yoshida, Tasuku Urita, Tomoaki Kitahara, Kengo Kadoya, Taiki Nabekura, Yuki Moriyama, Shinichi Okazumi

**Affiliations:** 1 Department of Surgery, Toho University Sakura Medical Center, 564-1 Shimoshizu, Sakura, Chiba, 285-8741 Japan; 2 Department of Surgical Pathology, Toho University Sakura Medical Center, Chiba, Japan

**Keywords:** Signet-ring cell carcinoma, Appendiceal cancer, Intestinal ganglioneuromatosis

## Abstract

**Background:**

Primary cancer of the appendix, especially signet-ring cell carcinoma, is an uncommon disease, and it is rarely suspected before surgery. Diffuse intestinal ganglioneuromatosis that is not associated with neurofibromatosis-1 or multiple endocrine neoplasia 2b is also rare. The most frequent symptoms caused by it are changes in bowel habits, abdominal pain, and occlusive episodes.

**Case presentation:**

The patient was a 48-year-old woman who had a month-long history of chronic abdominal pain, fullness, constipation, and diarrhoea. Enhanced computed tomography showed a 100-mm irregular swelling in the appendix and thickening of the appendiceal wall with cystic dilatation. Based on a preoperative diagnosis of appendiceal cancer, the patient underwent laparoscopic ileocecal resection with D3 lymph node dissection. Pathological diagnosis revealed a signet-ring cell carcinoma of the appendix with ganglioneuromatosis. The patient completed four courses of capecitabine plus oxaliplatin (CAPEOX) as postoperative adjuvant chemotherapy, and 23-month postoperative outcome was noneventful without recurrence.

**Conclusion:**

We report a signet-ring cell carcinoma of the appendix that was detected early because of its presence with ganglioneuromatosis.

## Background

Primary cancer of the appendix is an uncommon disease, and it is rarely suspected before surgery [1, 2]. Most patients are accidentally diagnosed during surgical procedures. This cancer type is usually diagnosed late when peritoneal dissemination has already occurred. Moreover, colonoscopy rarely detects appendiceal cancer [3]. According to a report published by the National Cancer Institute using the Surveillance, Epidemiology, and End Results database, appendiceal neoplasms account for approximately 0.4% of gastrointestinal tumours [4, 5]. Primary signet-ring cell carcinoma of the appendix is an exceedingly rare entity. Patients with signet-ring cell carcinoma of the appendix have greater tumour extension at diagnosis and have a significantly worse survival rate than patients with other types of tumour histology [5, 6].

Intestinal ganglioneuromatosis (IGNM) is also a rare neoplastic condition that is characterised by marked proliferation of ganglion cells, Schwann cells, and nerve fibres in the bowel wall [7]. Most neurological tumours that occur in the gastrointestinal tract are neurofibromas, which are associated with neurofibromatosis type 1 (NF1, so-called von Recklinghausen’s disease) and multiple endocrine neoplasia (MEN) type 2b. The most frequent symptoms caused by these tumours are changes in bowel habits, abdominal pain, and occlusive episodes.

Although colonic cases are even rarer, there is no reported case of signet-ring cell carcinoma of the appendix against the background of diffuse IGNM. Here, we report a case of a signet-cell carcinoma of the appendix that was detected early by examining the patient’s abnormal bowel movements and bloating, which are typical symptoms of IGNM.

## Case presentation

A 48-year-old woman was referred from primary care to our hospital. The patient had a month-long history of chronic abdominal pain, fullness, constipation, and diarrhoea; was diagnosed with irritable bowel syndrome by her family doctor; and was treated with ramosetron hydrochloride. The patient had a history of mild depression, but no other comorbidities. Her abdomen was soft and flat, with no peritoneal signs.

**Fig. 1:**
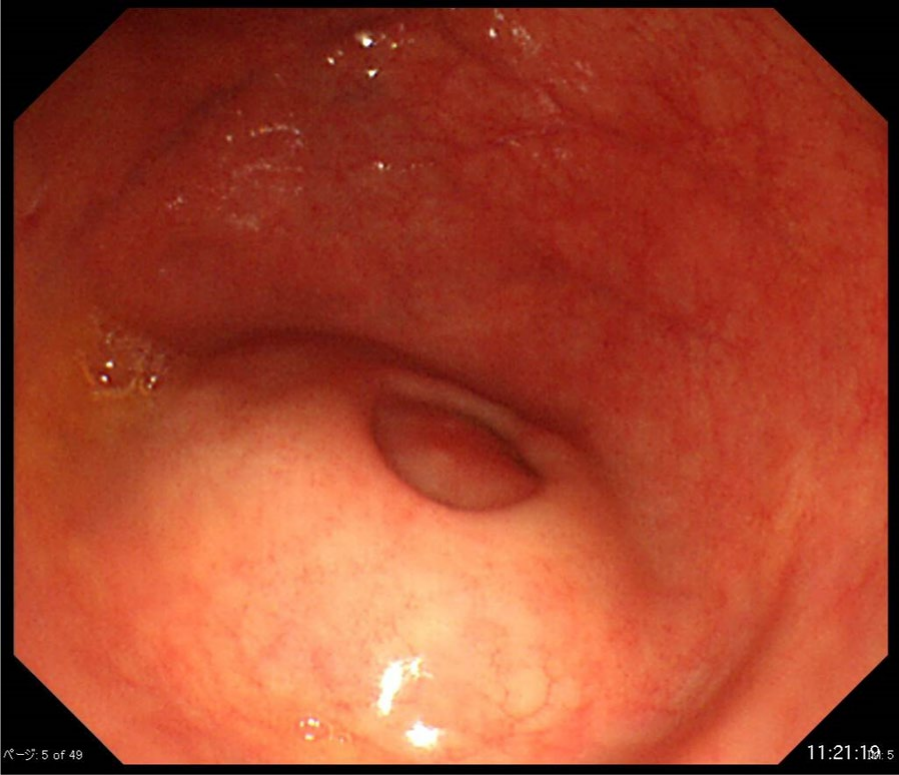
Endoscopic appearance of the appendix opening in the cecum on colonoscopy. The submucosal tumour was soft, with a positive cushion sign when we used forceps to compress the tumour

**Fig. 2:**
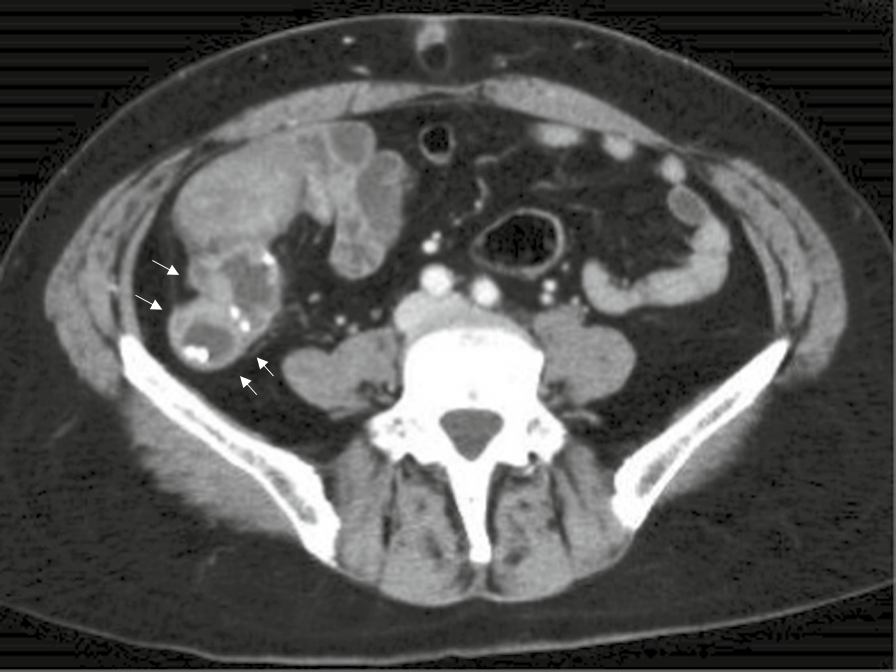
Axial images of contrast-enhanced computed tomography showing an irregular appendix (white arrows) swelling and thickening of the appendix wall with cystic dilation. Calcified lesions are also found inside the appendix

Laboratory data were as follows: white blood cell count, 7250/μL; C-reactive protein level, 0.98 mg/dL; carbohydrate antigen 19-9 level, 2.9 U/mL (normal < 37.0 U/mL); and carcinoembryonic antigen level, 2.1 ng/mL (normal < 5.0 ng/mL). Colonoscopy revealed 30-mm submucosal tumour-like findings in the cecum, which was probably the appendix opening (Fig. 1). No biopsy was performed. Enhanced computed tomography showed a 100-mm irregular swelling in the appendix and thickening of the appendiceal wall with cystic dilatation (Fig. 2).

**Fig. 3:**
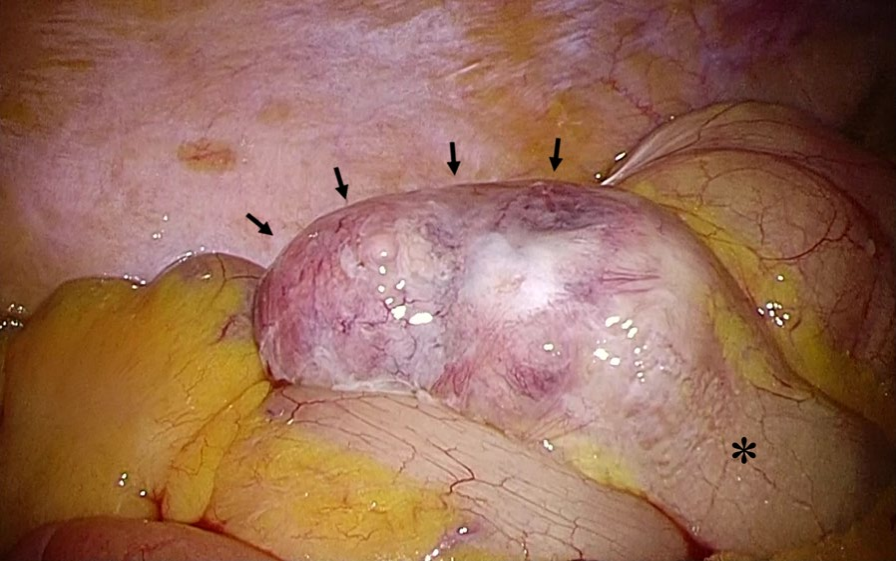
Intraoperative findings of the appendix. The appendix (black arrows) was dilated, and the border with the cecum (*) was unclear

Laparoscopic ileocecal resection with D3 lymph node dissection was performed with a preoperative diagnosis of appendiceal cancer. Laparoscopic findings included an irregular swelling of the appendix and a partial white discoloration on the appendiceal serosa surface; however, no invasion of surrounding tissues was observed. The appendiceal tumour was expansive, meandering, and thickened. Although a tumour-like mass was noted on the proximal side of the appendix, its boundary was unclear, and no tumour was found to form a clear nodule (Fig. 3). The appendiceal tumour in the resected specimen measured 80 × 60 × 40 mm.

**Fig. 4:**
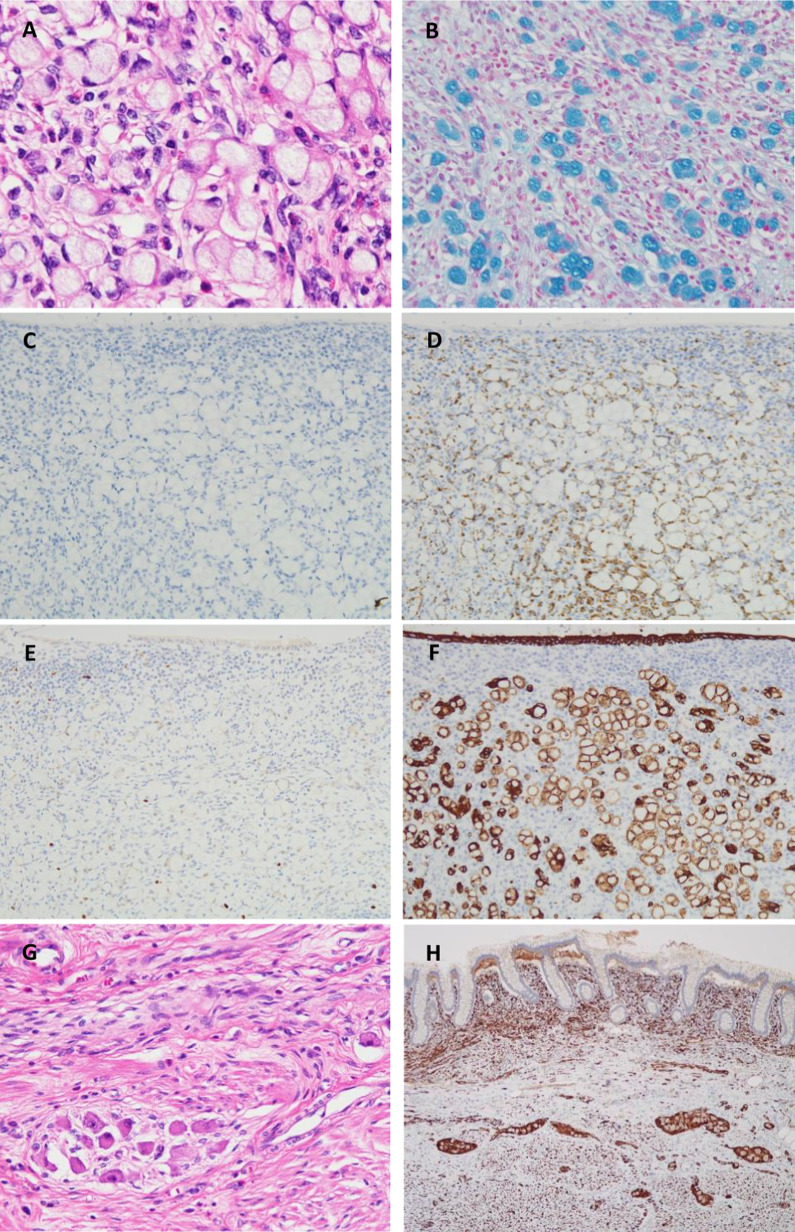
Histopathological examination. Proliferation of atypical cells, mainly signet-ring-like cells, in the appendix mucosa is observed using haematoxylin–eosin staining (**A**). Abundant mucus is confirmed using alcian blue staining (**B**). Immunohistochemical staining for chromogranin A (**C**) and synaptophysin (**D**) are negative in the cytoplasm of tumour cells. The immunoactivity for synaptophysin reveals nerve fibre proliferation in the mucosa of appendix. Immunohistochemical staining for insulinoma-associated protein 1, which is nuclear marker of neuroendocrine differentiation, is negative in the tumour cells (**E**). Immunostaining for CK (AE1/AE3) highlights the cytoplasm of the tumour cells and normal epithelium (**F**). Proliferation of spindle-shaped cells from the lamina propria to the lower layer in the colon is noted (**G**), and the neural marker (CD56) is positive (**H**)

Histologically, atypical cells mainly composed of signet-ring-like cells were found to proliferate solidly and invasively in the appendiceal mucosa, and they reached the subserosa (Fig. 4A). The tumour cells contained abundant mucus that was stained using alcian blue, and the infiltration was prominent in and around the nerves (Fig. 4B). Immunostaining results were as follows: CD56 (NCAM) (−), S-100 (−), chromogranin A (CGA) (−), synaptophysin (−), insulinoma-associated protein 1 (INSM1) (−), CEA (Mo) (+), CK (AE1/AE3) (+), CK7 (±), and CK20 (+). Tumour differentiation into neuroendocrine cells was unclear (Fig. 4C–F); thus, the tumour was considered a signet-ring cell carcinoma. No tumour invasion was observed in the veins and lymph vessels, and no metastases were noted in the lymph nodes. Additionally, spindle-shaped cells were observed to spread mainly in the mucosa from the appendix to the ascending colon continuously and from the submucosa to the muscularis depending on the region (Fig. 4G). Immunostaining showed that spindle-shaped cells were positive for neural markers, and the results were as follows: CD117 (c-kit) (−), CD34 (−), SMA (−), Desmin (−), S-100 (+), CD56 (NCAM) (+), CGA (±), synaptophysin (+) and Ki-67 (MIB-1) (low), indicating a ganglioneuromatosis (Fig. 4H).

After several days of postoperative treatment, the patient recovered well and did not have any abdominal pain, fullness, and diarrhoea. The patient agreed to four courses of capecitabine plus oxaliplatin (CAPEOX) as postoperative adjuvant chemotherapy for high-risk stage IIA (T3, N0) appendiceal cancer. Twenty-three months after surgery, the patient is alive and in good condition without recurrence.

## Discussion

McGory et al. reported that appendiceal signet-ring cell carcinoma has a high prevalence (60%) of distant metastases and has a meagre 5-year survival rate (18%). Furthermore, signet-ring carcinoma has a lower 5-year survival rate than other histological types, even if the cancer is excised in a localised state [6].

IGNM is a rare benign tumour that is characterised by an abnormal proliferation of ganglion cells, nerve fibres, and Schwann cells in the enteric nervous system [7]. IGNM can be found in any part of the gastrointestinal tract. The clinical presentation of IGNM is variable and depends on the location and extent of the lesions and their effect on gastrointestinal motility. The prevalent symptoms of IGNM are abdominal pain, constipation or diarrhoea, bleeding, changes in bowel habits, and intestinal obstruction owing to stricture formation [8, 9]. Generally, conservative treatment is ineffective for diffused IGNM, and complete resection is needed in symptomatic cases.

In our case, because IGNM coexisted and there were symptoms such as diarrhoea, constipation, and abdominal bloating, an appendiceal tumour was diagnosed based on gastrointestinal examination findings. The symptoms disappeared after the appendix and dilated intestine were surgically resected.

Appendiceal cancer is often detected at an advanced stage or at a stage of peritoneal dissemination. In our case, the patient was diagnosed with locally advanced cancer but with no peritoneal dissemination at the time of surgery, and no lymph node metastasis was detected during pathological examination of the resected specimen. Given the rarity of primary appendiceal cancer, especially signet-ring cell carcinoma, there are no randomised data regarding the efficacy of chemotherapy following the resection of the localised tumour. According to the NCCN guidelines, it is recommended that appendiceal adenocarcinomas be managed as per the guidelines for colon cancer, and adjuvant treatment with 5-FU-based chemotherapy is considered. In a study by Hugen et al., adjuvant chemotherapy for signet-ring cell carcinoma of the colon was associated with improved survival [10]. Among patients with signet-ring cell appendiceal tumours who underwent complete cytoreduction, adjuvant chemotherapy, including CAPEOX regimen, seemed to have significant benefit for survival [11]. The patient received postoperative adjuvant chemotherapy with four cycles of CAPEOX, similar to the treatment approach for colorectal cancer. The patient is currently well without disease recurrence.

To our best knowledge, this is the first reported case of signet-ring cell carcinoma of the appendix combined with diffuse IGNM. Coincidental development of ganglioneuromatosis and adenocarcinoma in the colorectal region has been reported previously. In 1981, Snover et al. were the first to report a case of diffuse ganglioneuromatosis associated with a poorly differentiated caecal adenocarcinoma [12]. The underlying molecular mechanisms remain unclear. When Qiao et al. experienced a case of colon adenocarcinoma associated with diffuse ganglioneuromatosis, they suggested that glial cell line-derived neurotrophic factor (GDNF) and neurturin (NTN) expression in adenocarcinoma cells may play an important role in the pathogenesis of IGNM [13]. GDNF and its receptor components, GDNF family receptor alpha-1 (GFRA1) and RET receptor tyrosine kinase, contribute to the development of the enteric nervous system. GDNF as well as NTN were expressed at high levels in adenocarcinoma cells. On the other hand, expression of GFRA1 and RET was detectable in proliferating ganglion cells and glial cells. Additionally, considering cancers that are more common in the general population, and NF1 cohort, elevated risk for colon cancer was found [14]. Among the patients diagnosed as a diffuse IGNM associated with NF1, somatic mutations in NF1 gene related with RAS pathway may be implicated in the development of colorectal cancer [15]. Although no association was identified between ganglioneuromatosis and the appearance of signet-ring cell carcinoma preoperatively in our case, in retrospect, the clinical manifestations of ganglioneuromatosis led to the early detection of appendiceal cancer and a good prognosis of this malignant disease.

## Conclusion

This report showed a rare case of diffuse IGNM not associated with NF1 or MEN2b. Although the prevalence of ganglioneuromatosis is low, this condition should be considered in the differential diagnosis of intestinal mass in adults. We also found that signet-ring cell carcinoma of the appendix may be discovered against the background of ganglioneuromatosis. We reported a signet-ring cell carcinoma of the appendix, which is more likely to be detected as an advanced cancer, in a localised condition because of its presence with ganglioneuromatosis.

## Data Availability

Not applicable.

## Abbreviations

IGNM: Intestinal ganglioneuromatosis
NF1: Neurofibromatosis type 1
MEN: Multiple endocrine neoplasia
CGA: Chromogranin A
CAPEOX: Capecitabine plus oxaliplatin
GDNF: Glial cell line-derived neurotrophic factor
NTN: Neurturin
GFRA1: GDNF family receptor alpha-1
